# Annual consumption of parenteral antibiotics in a tertiary hospital of Nepal, 2017–2019: a cross-sectional study

**DOI:** 10.5588/pha.21.0043

**Published:** 2021-11-01

**Authors:** P. Baral, K. Hann, B. Pokhrel, T. Koirala, R. Thapa, S. M. Bijukchhe, M. Khogali

**Affiliations:** 1 Department of Pharmacy, Modern Technical College, Sanepa, Lalitpur, Nepal; 2 Sustainable Health System, Freetown, Sierra Leone; 3 Department of Paediatrics, Patan Academy of Health Sciences, Lalitpur, Nepal; 4 Dasharathpur Primary Health Centre, Department of Health Services, Ministry of Health and Population, Surkhet, Nepal; 5 Department of Pharmacy, Patan Academy of Health Sciences, Lalitpur, Nepal; 6 UNICEF/UNDP/World Bank/WHO Special Programme for Research and Training in Tropical Diseases (TDR), World Health Organization, Geneva, Switzerland

**Keywords:** ATC/DDD, AWaRe, operational research, SORT IT

## Abstract

**SETTING:**

Patan Hospital, a tertiary care hospital in Lalitpur District, Nepal.

**OBJECTIVES:**

To describe the annual parenteral antibiotic consumption in 1) defined daily dose (DDD) and DDD per 100 admissions; 2) calculate DDD per 100 admissions and proportions by pharmacological subgroup, chemical subgroup and AWaRe categories; and 3) describe patient expenditure on parenteral antibiotics as a proportion of the total patient expenditure on drugs and consumables between 2017 and 2019.

**DESIGN:**

This was a cross-sectional study.

**RESULTS:**

Total DDD of parenteral antibiotics increased by 23% from 39,639.7 in 2017 to 48,947.7 in 2019. DDD per 100 admissions increased by 10% from 172.1 in 2017 to 190.2 in 2019. Other beta-lactam antibacterials comprised the most frequently consumed pharmacological subgroup. The chemical substance most often consumed was ceftriaxone, with an increasing trend in the consumption of vancomycin and meropenem. Parenteral antibiotics in ‘Watch’ category were the most consumed over the study period, with a decreasing trend in ’Access’ and increasing trend in ‘Reserve’ categories.

**CONCLUSION:**

We aimed to understand the consumption of parenteral antibiotics at a tertiary care hospital and found that Watch antibiotics comprised the bulk of antibiotic consumption. Overconsumption of antibiotics from the ‘Watch’ and ‘Reserve’ categories can promote antimicrobial resistance; recommendations were therefore made for their rational use.

## Abstract (FR)

**CONTEXTE:**

Hôpital de Patan, un hôpital de soins tertiaires du District de Lalitpur, Népal.

**OBJECTIFS:**

Décrire la consommation annuelle d’antibiotiques parentéraux en 1) dose définie journalière (DDD) et DDD pour 100 admissions ; 2) calculer la DDD pour 100 admissions et les pourcentages par sous-groupe pharmacologique, sous-groupe chimique et catégories de la classification AWaRe ; et 3) décrire les dépenses des patients en antibiotiques parentéraux en tant que proportion du total des dépenses des patients en médicaments et consommables de 2017 à 2019.

**MÉTHODE:**

Il s’agissait d’une étude transversale.

**RÉSULTATS:**

La DDD totale d’antibiotiques parentéraux a augmenté de 23%, de 39 639,7 en 2017 à 48 947,7 en 2019. La DDD pour 100 admissions a augmenté de 10%, de 172,1 en 2017 à 190,2 en 2019. Les autres bêtalactamines étaient le sous-groupe pharmacologique le plus consommé. La substance chimique la plus consommée était la ceftriaxone, avec une tendance à la hausse de la consommation de vancomycine et de méropénème. Les antibiotiques parentéraux du groupe « antibiotiques à utiliser sélectivement » étaient les plus consommés pendant la période d’étude, avec une tendance à la baisse de ceux du groupe « antibiotiques dont l’accessibilité est essentielle » et une tendance à la hausse de ceux du groupe « antibiotiques de réserve ».

**CONCLUSION:**

Nous avons cherché à comprendre la consommation d’antibiotiques parentéraux dans un hôpital tertiaire et avons observé que les « antibiotiques à utiliser sélectivement » représentaient la majorité de la consommation d’antibiotiques. La surconsommation d’« antibiotiques à utiliser sélectivement » et d’« antibiotiques de réserve » peut favoriser la résistance antimicrobienne. Des recommandations ont donc été émises pour un usage raisonné de ces antibiotiques.

Antimicrobial resistance (AMR) is a global public health threat.^1^–^4^ To date, AMR has been responsible for 700,000 deaths per year worldwide, and is expected to reach 10 million per year by 2050.^5^ The potential of AMR to increase morbidity and mortality is even higher in low- and middle-income countries (LMICs), where the burden of infectious disease is higher.^6^ In addition to its serious health implications, AMR has a significant impact on healthcare costs.^7^ Inappropriate antibiotic consumption has been identified as the main driver of AMR.^1^,^4^,^8^ Globally, the consumption of antibiotics has increased by 67% between 2000 and 2015,^1^,^2^,^8^ and almost half of all antibiotic consumption in healthcare is considered inappropriate.^9^ To monitor the consumption of antimicrobials and promote the prudent use of antibiotics, the WHO developed a methodology for antimicrobial consumption surveillance using a metric, the defined daily dose (DDD), as per the anatomical, therapeutic and chemical classification (ATC) system.^10^ DDD is the assumed average maintenance dose per day of antimicrobial substances used for its main indication in adults. In addition, the WHO has classified antibiotics into three categories as AWaRe: ‘Access’ (antibiotics needed for common infections that should be available and accessible), ‘Watch’ (broad-spectrum antibiotics that should be used with caution because of their high potential to develop resistance) and ‘Reserve’ (antibiotics that should be reserved for the treatment of multi-drug-resistant infections and used only when other alternatives fail).^11^

While consumption of all types of antibiotics should be rational, the wise use of parenteral antibiotics is particularly important, as this type of antibiotics should be preserved for patients who are unable to swallow or absorb oral antibiotics, such as those who are critically ill.^12^ Parenteral drugs are non-oral drugs, administered by injection; these can thus achieve therapeutic concentrations reliably and rapidly. However, antibiotics administered parenterally are more expensive than their oral counterparts and more costly to administer.^13^ Anecdotal evidence suggests that there is overconsumption of parenteral antibiotics in Patan Hospital, Lagankhel, Lalitpur District, Nepal. To date, there has been no formal assessment to prove or refute this observation. One possible reason could be that the majority of hospitals in Nepal, including Patan Hospital neither have formal antibiotic stewardship programme nor hospital-specific guidelines or protocols for the rationale use of antibiotics.

Therefore, we aimed to assess the trend and pattern of parenteral antibiotic consumption and related patient expenditure in Patan Hospital. Specific objectives were to describe annually 1) parenteral antibiotic consumption in DDD and DDD per 100 admissions; 2) parenteral antibiotic consumption in DDD per 100 admissions and proportions by pharmaceutical subgroup (ATC3), chemical subgroup (ATC5), and AWaRe categories; and 3) patient expenditure on parenteral antibiotics as a proportion of total patient expenditure on all drugs and consumables utilised by inpatients for the period 2017 to 2019.

## METHODS

### Study design

This was a cross-sectional study.

### Setting

Nepal is a small landlocked country located between India and China with a population of 30.2 million, 40% of which live below the poverty line.^14^ There is an increasing burden of AMR in Nepal, with published literatures showing widespread distribution of resistant bacterial infections.^3^,^15^ The Government of Nepal drafted the National Antibiotic Treatment Guidelines in 2014 to promote the rational use of antimicrobial agents across all clinical settings in the country, with the aim to 1) prevent unnecessary use and drug resistance; 2) improve safety by avoiding unnecessary drug toxicity; and 3) reduce costs.^16^ The guidelines provide a list of essential medicines, which includes relatively safe antibiotics that are effective and low cost, with few side effects.

Patan Hospital, one of the largest in Nepal, is a 640-bed, autonomous, not-for-profit, tertiary-level hospital in Lalitpur District of Province 3, serving mostly people in the Kathmandu valley.

### Drug procurement system

The hospital procures drugs and consumables on an annual basis by inviting bids through a tendering system in accordance with Nepalese government regulations,^17^ based on an annual budget which is increased every year by 10%. Decisions on volumes and classes of drugs are based on previous annual consumption of drugs. All drugs must be purchased from manufacturers who are WHO good manufacturing practice (GMP) certified.^18^

### Drug distribution system

For inpatients, the Pharmacy Department distributes drugs using the ward stock system, where drugs are managed at the ward level, and unit dose system, where doses of a specific drug are prescribed for a certain patient at a specific time.^19^ Patients pay for any drugs consumed and other healthcare costs upon discharge from the hospital. Prices for patient purchasing are uniformly set at 16% above the procurement cost

### WHO ATC/DDD classification system

Consumption was calculated using the WHO methodology for surveillance of antimicrobial substances, and based on the ATC/DDD classification using both anatomical, therapeutic, pharmacological (ATC3) and chemical properties (ATC5).^10^

DDD, which is a statistical measure of drug consumption, should not be confused with the therapeutic dose or prescribed daily dose; it often differs from the dose actually prescribed by a physician.^10^ DDDs are often presented in terms of units that control for population size differences. Due to lack of information on the hospital catchment area, consumption was presented as the number of DDDs per 100 admissions.

### Study population

The study included parenteral antibiotics for systemic use (ATC J01) consumed by patients admitted over 3 years, from 2017 to 2019. Anti-TB drugs, oral antibiotics and topical antibiotics were excluded.

### Data sources and variables

Data on the number of admissions, number of parenteral antibiotics and the total expenditure for drugs and consumables dispensed for inpatient consumption were extracted from an electronic database maintained in the Hospital Management Information System (MariaDB 10.3 HealthyLife; https://go.mariadb.com/) from the Medical Records Section and Pharmacy Department into Microsoft Excel (Microsoft, Redmond, WA, USA). Variables collected included international non-proprietary names, substance strength and unit, annual drug unit price for patients and quantity of units dispensed for inpatients each year. ATC5 and ATC3 codes, DDD values and AWaRe category were manually assigned in Microsoft Excel by two data clerks and validated by the principal investigator.

### Data analysis

Consumption was calculated per year by frequency of DDD and DDD per 100 admissions (annual DDD divided by total patient admissions and multiplied by 100). Patient expenditure was calculated by multiplying quantity of units dispensed for inpatients by the drug unit price for patients per year. Patient expenditure on parenteral antibiotics was calculated as a proportion of total patient expenditure on all drugs and consumables. Drug unit price and patient expenditure were converted from Nepali rupees to US dollars using the mean exchange rate for each year under review. Summary measures (frequency and percentage) were used to report on categorical variables.

### Ethics approval

Ethics approval was obtained from the Institutional Review Board of Patan Academy of Health Sciences, Lalitpur, Nepal (Ref: out2009181448l), and the Ethics Advisory Group of the International Union Against Tuberculosis and Lung Disease, Paris, France (EAG number: 12/2020).

## RESULTS

### Annual total patient admissions and parenteral antibiotic consumption in defined daily dose

**FIGURE 1:**
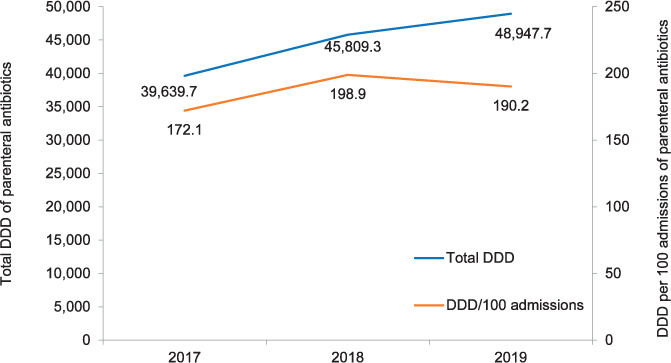
Annual consumption of parenteral antibiotics in terms of DDD in Patan Hospital, Lalitpur, Nepal, 2017–2019. DDD = defined daily dose.

The total number of patients admitted to the hospital was respectively 23,032, 25,976 and 25,737 in 2017, 2018 and 2019. Annual consumption of parenteral antibiotics is shown in Figure 1. The total DDD increased from 39,639.7 in 2017 to 45,809.3 in 2018, and reached 48,947.7 in 2019. The increase in total DDD was higher between 2017 and 2018 than between 2018 and 2019. The DDD per 100 admissions increased from 172.1 in 2017 to 198.9 in 2018, and reduced to 190.2 in 2019.

### Annual parenteral antibiotic consumption by pharmacological subgroup

Other β-lactam antibacterials (J01D), β-lactam antibacterials, penicillins (J01C) and other antibacterials (J01X) pharmacological subgroups were the most consumed parenteral antibiotics by pharmacological subgroup (ATC3) over the 3-year period (Table 1).

**TABLE 1:** Annual parenteral antibiotic consumption in terms of DDD per 100 admissions and proportions (%) by pharmacological subgroup (ATC3) in Patan Hospital, Lalitpur, Nepal, 2017–2019

Pharmacological subgroup	ATC3	Parenteral antibiotic consumption DDD per 100 admissions					

		2017		2018		2019	

		*n*	(%)	*n*	(%)	*n*	(%)
Other β-lactam antibacterials	J01D	56.9	(33)	75.8	(38)	61.9	(33)
β-lactam antibacterial penicillins	J01C	48.9	(28)	52.4	(26)	57.8	(30)
Other antibacterials	J01X	31.5	(18)	36.6	(18)	35.9	(19)
Aminoglycoside antibacterials	J01G	14.3	(8)	18.0	(9)	16.1	(9)
Quinolone antibacterials	J01M	16.7	(10)	13.8	(7)	13.2	(7)
Macrolides, lincosamides and streptogramins	J01F	3.8	(2)	1.5	(1)	4.9	(3)
Amphenicols	J01B	0.1	(0)	0.8	(0)	0.4	(0)
Total		172.1	(100)	198.9	(100)	190.2	(100)

DDD = defined daily dose; ATC3 = Anatomical Therapeutic Chemical classification system, level 3 (pharmacological subgroup).

### Annual parenteral antibiotic consumption by chemical subgroup

The most common parenteral antibiotics admissions by chemical subgroup (ATC5) consumed over the 3-year period were ceftriaxone (J01DD04), followed by cloxacillin (J01CF02) and metronidazole (J01XD01) (Table 2). The consumption of ceftriaxone increased from 51.5 (30%) in 2017 to 70.1 (35%) in 2018, and decreased to 57.3 (30%) in 2019. The consumption of cloxacilin remained the same (at 30.4) in 2017 and 2018, but slightly increased to 31.8 in 2019. However, its proportion of all parenteral antibiotics consumed decreased from 18% in 2017 to 15% in 2018, and rose to 17% in 2019. For metronidazole, consumption increased from 21.8 (13%) in 2017 to 25.0 (13%) in 2018, and fell to 19.6 (10%) in 2019. There was an increasing trend in consumption of vancomycin, linezolid and meropenem over the study period.

**TABLE 2:** Annual parenteral antibiotic consumption in terms of DDD per 100 admissions and proportions (%) by chemical subgroup (ATC5) in Patan Hospital, Lalitpur, Nepal, 2017–2019

Chemical substance	ATC5	Parenteral antibiotic consumption DDD per 100 admissions					

		2017		2018		2019	

		*n*	(%)	*n*	(%)	*n*	(%)
Ceftriaxone	J01DD04	51.5	(30)	70.1	(35)	57.3	(30)
Cloxacillin	J01CF02	30.4	(18)	30.4	(15)	31.8	(17)
Metronidazole	J01XD01	21.8	(13)	25.0	(13)	19.6	(10)
Piperacillin + beta-lactamase inhibitor	J01CR05	17.9	(10)	21.4	(11)	25.7	(14)
Amikacin	J01GB06	9.4	(6)	12.1	(6)	9.9	(5)
Vancomycin	J01XA01	6.4	(4)	7.8	(4)	10.4	(6)
Ciprofloxacin	J01MA02	6.0	(4)	6.0	(3)	5.5	(3)
Gentamicin	J01GB03	4.9	(3)	5.9	(3)	6.2	(3)
Levofloxcin	J01MA12	8.3	(5)	4.8	(2)	5.2	(3)
Cefotaxime	J01DD01	3.4	(2)	3.2	(2)	1.8	(1)
Ofloxacin	J01MA01	2.4	(1)	3.0	(2)	2.5	(1)
Ornidazole	J01XD03	2.4	(1)	2.5	(1)	3.5	(2)
Azithromycin	J01FA10	3.8	(2)	1.5	(1)	4.9	(3)
Meropenem	J01DH02	0.8	(0)	1.4	(1)	1.7	(1)
Imipenem + cilastatin	J01DH51	1.3	(1)	1.2	(1)	1.2	(1)
Linezolid	J01XX08	0.2	(0)	0.9	(0)	1.5	(1)
Chloramphenicol	J01BA01	0.1	(0)	0.8	(0)	0.4	(0)
Ampicillin	J01CA01	0.6	(0)	0.6	(0)	0.3	(0)
Colistin	J01XB01	0.8	(1)	0.4	(0)	0.9	(1)
Total		172.1	(100)	198.9	(100)	190.2	(100)

DDD = defined daily dose; ATC5 = Anatomical Therapeutic Chemical classification system, level 5 (chemical substance).

### Antibiotic consumption by AWaRe category

The most commonly used parenteral antibiotics by AWaRe category were those from the ‘Watch’ category, followed by the ‘Access’ category. The ‘Watch’ category that were most frequently used included ceftriaxone, and the combination of piperacillin and β-lactamase inhibitor; cloxacillin and metronidazole were the most frequently consumed ‘Access’ group antibiotics. Consumption of ‘Reserve’ category antibiotics increased from 7.4 (3.7%) in 2018 to 9.3 (4.9%) in 2019. The consumption of meropenem increased two-fold, while that of linezolid increased by more than five times in 2019 as compared to that of 2017 (Table 3).

**TABLE 3:** Annual parenteral antibiotic consumption in terms of DDD per hundred admissions and proportions (%) by AWaRe classification in Patan Hospital, Lalitpur, Nepal, 2017–2019

WHO AWaRe Category	Parenteral antibiotic consumption DDD per 100 admissions					

	2017		2018		2019	

	*n*	(%)	*n*	(%)	*n*	(%)
Access	69.5	(40.4)	77.3	(38.9)	71.7	(37.7)
Watch	92.6	(53.8)	114.1	(57.4)	109.2	(57.4)
Reserve	10.0	(5.8)	7.4	(3.7)	9.3	(4.9)

DDD = defined daily dose; WHO AWaRe = World Health Organization Access, Watch, and Reserve classification of antibiotics.

### Patient expenditure on parenteral antibiotics

**FIGURE 2.**
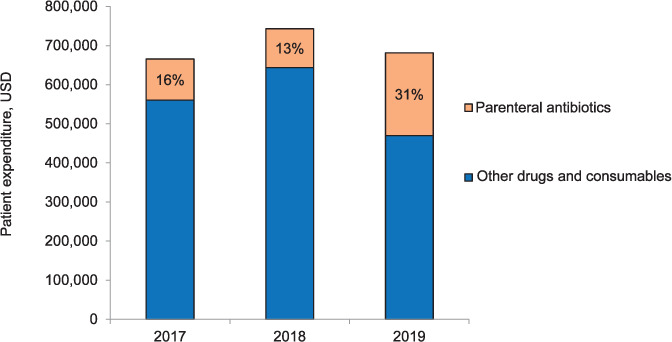
Proportion of patient expenditure on parenteral antibiotics of total patient expenditure on all drugs and consumables utilised for management of inpatients in Patan Hospital, Lalitpur, Nepal, 2017–2019. USD = US dollar.

Total expenditure on parenteral antibiotics for the years 2017, 2018 and 2019 was respectively 105,110, 99,079 and 211,453 in USD. Annual proportion of patient expenditure for parenteral antibiotics consumed of the total patient expenditure on all drugs and consumables used for inpatients decreased from 16% in 2017 to 13% in 2018, and then increased almost two-fold from baseline to 31% in 2019 (Figure 2).

## DISCUSSION

This study showed an increase in the overall consumption of parenteral antibiotics in DDD and in DDD per 100 hospital admissions from 2017 to 2019. However, the latter decreased between 2018 and 2019, which can be attributed to the decrease in the total number of admissions. Other β-lactam antibacterials, β-lactam antibacterial penicillins and other antibacterials were the three most commonly consumed pharmacological subgroups (ATC3), while ceftriaxone, cloxacillin and metronidazole were the three chemical substances (ATC5) most frequently used. The highest proportion of consumption of parenteral antibiotics was in the ‘Watch’ category, with a decreasing proportion of ‘Access’ and increasing proportion in the ‘Reserve’ category over time. The proportion of patient expenditure on parenteral antibiotics doubled in 2019 as compared to 2017.

Our study findings have important implications for health policy and practice. First, the increase in overall volume of consumption may be due to differences in patient characteristics, their need for parenteral antibiotics, annual variation on the mean duration of hospital stay, antibiotic policies and physician training, which may change with time.^20^ Effective interdisciplinary education, cooperation between physicians and microbiologists and modern diagnostic methods to differentiate viral from bacterial illnesses may prevent the overconsumption of antibiotics.^21^

Second, the ATC5 drug most frequently consumed over the 3-year period was ceftriaxone. Similar high consumption of ceftriaxone was also seen in another tertiary hospital in Nepal.^22^ Preference for ceftriaxone may be due to its high antibacterial potency, wide spectrum of activity and low potential of toxicity.^23^ In contrast, ampicillin was the most commonly used antibiotic in adult internal medicine ward of one of the largest tertiary care teaching hospitals in western Nepal.^24^ Similar high consumption of ampicillin was also found in the intensive care unit of the same hospital.^25^ We found an increasing trend in the consumption of vancomycin and meropenem, which is similar to that observed in tertiary and secondary hospitals in Singapore.^26^ This increase may be due to the emergence of extended spectrum β-lactamase-producing bacteria and methicillin-resistant staphylocci.^27^ Studies from Nepal have shown a pattern of increasing antimicrobial resistance to commonly used antibiotics.^3^,^28^ Surveillance on sensitivity patterns of bacterial pathogens isolated from clinical samples can help to limit the use of such broad-spectrum antibiotics.^29^ Establishing hospital guidelines for antibiotic prescribing, education programmes, use of antibiotic order forms, introduction of electronic prescribing and requiring approvals from infectious disease specialists before using broad-spectrum antibiotics are strategies to prevent irrational use and overconsumption.^30^

Third, by AWaRe category, we observed that ‘Watch’ category drugs were most frequently consumed, the proportion of ‘Access’ category drugs consumed decreased, while ‘Reserve’ category drugs increased; this is in line with findings from China.^31^ This finding may reflect changing patterns of resistance among bacterial pathogens isolated from patients in Patan Hospital.^32^ This is quite alarming, as the overuse of ‘Watch’ and ‘Reserve’ categories of antibiotics can favour the selection and spread of multidrug-resistant bacteria.^33^ Antibiotic stewardship programmes and effective infection prevention control measures can help to avoid the overuse of these groups of antibiotics. Similarly, decision-making for drug procurement should be informed by consumption trends, and should identify opportunities to address the issue of the increasing proportion of ‘Watch’ and ‘Reserve’ antibiotics procured and dispensed at the hospital.^34^

Fourth, patient expenditure on parenteral antibiotics doubled from 2017 to 2019. Antibiotic overuse related to non-existence of or non-adherence to antibiotic prescribing guidelines, potential increases in infectious illnesses among admitted patients and clinician preference of parenteral over oral antibiotics may cause increases in patient costs. Adherence to national antibiotic guidelines which promote the safe and effective use of antibiotics can help to limit the irrational prescribing pattern, and hence consumption and costs.^14^

This study had several strengths. First, as it was based on routinely collected data, it reflects the operational reality of antimicrobial consumption in one referral hospital in Nepal. Second, this is the first study from Nepal to use DDD per 100 hospital admissions as the unit for consumption. This unit is less influenced by the length of stay, as compared to DDD per 100 bed days, and more likely to correlate with the risk of AMR.^20^ Third, the standard definition and classification of parenteral antibiotics (ATC) was used, which allows for comparability of findings in other contexts. Fourth, this study followed the Strengthening of Reporting of Observational Studies in Epidemiology (STROBE) guidelines.^35^

This study also had some limitations. First, we measured antibiotics dispensed from the pharmacy, not those truly consumed by the admitted patients. Second, our data did not take into account potential multiple admissions resulting from intra-hospital transfers. This may have resulted in the overestimation of total admissions and underestimation of DDD per 100 admissions. Third, we could not assess parenteral antibiotic consumption per department as the pharmacy supplies some antibiotics using the unit dose system and does not record this as department-wise data.

In conclusion, this study highlighted the increase in overall consumption of parenteral antibiotics, with the highest proportion of antibiotics consumed throughout the study period in the ‘Watch’ category. A decrease in the proportion of ‘Access’ antibiotics consumed and increase in ‘Reserve’ category parenteral antibiotics were observed. Ceftriaxone was the most frequently consumed parenteral antibiotic, with rising trends in the consumption of vancomycin and meropenem. Recommendations were made to promote the rational use of parenteral antibiotics, including the development of hospital-specific guidelines on antibiotic use.
